# Training load influences gut microbiome of highly trained rowing athletes

**DOI:** 10.1080/15502783.2025.2507952

**Published:** 2025-05-21

**Authors:** B. Charlesson, J. Jones, C. Abbiss, P. Peeling, S. Watts, C.T. Christophersen

**Affiliations:** a Edith Cowan University, School of Medical and Health Science, Perth, Australia; b Western Australian Institute of Sport, Perth, Australia; c University of Western Australia, School of Human Sciences, Perth, Australia

**Keywords:** Gut health, exercise, short chain fatty acids, microbiota, transit time, diet quality

## Abstract

**Background:**

Despite the importance of the gut microbiome on physical performance and health, little is known on the impact of training on an athlete’s gut health.

**Objective:**

This study investigates the effect of training load on markers of gut health.

**Methods:**

Whole stool (24 h) samples were collected from 23 highly trained rowers (mean ± SD; age 19.2 ± 1.1 y; weight 80.1 ± 11.4 kg; height 1.83 ± 0.09 m) following periods of high (HT) and low training load (LT). The microbiome and short-chain fatty acid concentrations were characterized from the whole stool samples. Three-day weighted food records were used to determine diet quality (ADIcore), macronutrient, and fiber intakes during HT and LT.

**Results:**

By design, training duration (147%) and intensity (130%) were greater during (HT), compared with (LT) (*p* < 0.001). Carbohydrate, fat, protein, and fiber intake remained stable, but ADIcore was higher in HT (55 ± 10) compared with LT (49 ± 9; t(15) = 2.78, *p =* 0.014; CI: 1.34 to 10.155). Stool frequency (1.11 ± 0.47 vs 0.67 ± 0.76; *p =* 0.007) was lower in HT compared with LT, and a greater number of participants were unable to produce a stool sample during LT (8% vs 47%). Short chain fatty acid (SCFA), propionic (120.64 ± 30.06 mm vs 91.35 ± 34.91 mm; *p =* 0.007), and butyric acid (104.76 ± 50.02 vs 64.23 ± 22.05 mm, *p =* 0.003) concentrations were lower in HT compared with LT. Alpha diversity, Shannon–Wiener diversity index (3.43 ± 0.37 vs 3.67 ± 0.34, *p =* 0.09) was lower in HT than LT. The abundance of the dominant *Bacteroidia* was greater at HT compared to LT and ratio of firmicutes to *Bacteroidota* (*n* = 16, 1.31 ± 1.19 vs 4.29 ± 3.88, t(15) = -3.44, *p =* 0.04, CI = -4.82 to −1.13) was lower in HT compared to LT.

**Conclusion:**

Results of this study indicate that gut microbiome, SCFA concentrations, stool frequency, and diet quality vary between periods of high and low training load in athletes. The relationship between these factors and impact of such changes in gut health is currently unclear and warrants further investigation.

## 1. Background

An athlete’s ability to consume, digest, and absorb adequate nutrients has considerable impact on sports performance [1]. Consequently, gut health research in athletes predominantly focuses on improving nutrient digestion and absorption or the prevention and management of gastrointestinal (GI) symptoms [2,3]. Gut microbiome and the functions of the microbial communities that reside within the gastrointestinal tract of athletes is often overlooked. However, recent research has begun to examine the potential role of the gut microbiome in athlete health and performance [4].

Based upon the current literature, it appears that athletes have a different gut microbiota, when compared with the general population [5–8]. This includes: greater total short chain fatty acid concentrations; alpha diversity; an increased abundance of some bacteria, such as *Clostridiales*, *Roseburia*, *Lachnospiraceae,* and *Erysipelotrichaceae* [6]; and lower abundance of others (e.g. *Bacteroides* and *Lactobacillus* genus) [7]. Whilst compositional differences in the gut microbiomes of athletes and the general population are likely to be primarily related to differences in dietary intake [9], markers of fitness, including peak or maximal oxygen uptake, have also been correlated [6,10]. Differing microbiome composition also appear to link to functional outcomes within athlete populations. These include studies showing: improved ultra endurance performance with increased abundance of lactate metabolizing bacteria [11,12]; an association between training time and *Prevotella* abundance [13]; and observed variations in gut microbiome composition between athlete type (strength vs endurance), training phase (transition vs preparation) [10] and diet manipulations [14]. Although this cross-sectional research provides evidence to suggest that improving gut microbiome may benefit performance in athletes, there is currently limited longitudinal research and a lack of understanding regarding the factors influencing gut microbiome in athletes.

There is some evidence that markers of gut health may be impacted by exercise and training load [9,15–17]. In the general population, it has been found that transit times are quickened by the inclusion of running or cycling [18] and in endurance athletes increased intestinal motor activity has been observed with increased training intensity [19]. Additionally, accelerated colonic transit times, increased stool frequency and looser stool consistency have been shown during periods of high training compared with a rest period [20]. In the general population, transit time, stool frequency, and stool consistency have been linked to changes in the microbiome, including the increased abundance of *Bacteroides* and alpha diversity with slower transit times [21]. When exploring this topic therefore, a wider understanding of gut health, including transit time, stool consistency, and frequency in athletes, is essential for the identification of modifiable factors to elicit performance and health gain.

The present study aims to explore differences in markers of gut health (fecal microbiome and SCFA concentrations, stool frequency, and consistency) during two distinct phases of training, high training load (HT), and low training load (LT), in well trained elite rowing athletes. Detailed information on dietary intake, diet quality, and stool conditions are collated alongside the primary outcomes (microbiota and training load) to improve our understanding of the factors influencing gut health in athletes.

## 2. Methods and materials

### 2.1. Subject characteristics

Twenty-three, highly trained, national level rowing athletes [22], (mean ± SD; age 19.2 ± 1.1 y; weight 80.1 ± 11.4 kg; height 1.83 ± 0.09 m) from a state based, rowing program were recruited to participate in this study. Participants included 11 males (age 19.5 ± 0.9 y; weight; 88 ± 8.1 kg; height 1.90 ± 0.06 m) and 12 females (age 19.1 ± 1.1 y; weight 70.5 ± 6.2 kg; height 1.77 ± 0.05 m).

Four participants (4 males) withdrew from the study, during the LT due to COVID-19 restrictions. As a result, female participants account a greater proportion of the cohort during LT (63%) when compared to HT (52%).

Participants were excluded if they were currently smoking, pregnant or planning to become pregnant, breastfeeding, currently using pharmaceutical agents that could modify gut health (e.g. probiotics, antibiotics, eluxadoline, lubiprostone, and linaclotide), or had taken prebiotics in the past 6 weeks, a known diagnosis of other gastrointestinal illness (e.g. inflammatory bowel disease, malabsorption of any macronutrients, bowel resection, celiac disease), previous abdominal or gastrointestinal surgeries, severe mental health and sleep-related conditions (e.g. insomnia), renal or hepatic diseases, and/or other major medical illness, or any other disease, condition or habit that may interfere with completion of study.

### 2.2. Experimental design

Training load, dietary intake, and fecal samples were collected (described below) during 2 three-day study periods. The first study period was a high training phase (HT) and the second was at low training load (LT). HT occurring during an intense training period, three weeks prior to a targeted national competition; and LT occurring 7 days following the commencement of the off-season. Study periods were separated by 1 month.

#### 2.2.1. Training load

Participants were required to wear a heart rate monitor and global positioning systems (GPS) devices for all training, including running, cycling, rowing, and other structured training sessions over a three-day period. The heart rate monitor was started and stopped by participants and uploaded to training software (Training Peaks, Louisville, CO) for the purpose of calculating training duration and Training Stress Score® (TSS®). Uploaded data was reviewed by researchers and edits made using additional notes, e.g. modifications of start times, training modalities, and missing heart rate data. Training history and functional threshold heart rates were known for all participants prior to commencement of the study and used to inform TSS® calculation. TSS® that takes into account the duration and intensity to provide a single marker estimating the physiological stress created a training session.

#### 2.2.2. Dietary intake

Dietary intake was collected using a 3-day weighed food record. Participants recorded this using the Easy Diet Diary App. Upon completion, the food record was emailed to the primary researcher using the in-app functionality. The food record commenced 24-h prior to first stool sample collection and was completed at the end of the second 24-h stool sample collection period. Participant were asked to report use of supplement and medications. Three-day weighed food records were reviewed by primary researcher via face-to-face or phone interview with participant. Once verified, the food records were updated and analyzed in using Foodworks 10 software. Food records were also assessed for diet quality using the Athlete Diet Index following methodology outlined by Capling et al. [23] and core food rating was reported (ADIcore). Two participants did not complete the 3-day weighed food record during LT.

#### 2.2.3. Fecal samples

At each time point, two 24-h whole stool sample collections were completed. Participants were directed to collect all stools across the 24-h periods (0–3 samples). After the end of each 24-h collection period, samples were returned to the testing laboratory, placed in a portable freezer, transported frozen, and transferred into a −80°C freezer within 4 h. Samples remained frozen at −80°C until processed.

For each stool collection, participants were asked to record time of day and classify stool consistency using the Bristol Stool Chart. Participants were also asked to state if they perceived the stool sample as normal and comments were requested if participants stated the samples was abnormal. Each stool sample was collected individually and the whole stool sample remained cold using Techni Ice Heavy Duty Reusable Dry Ice Pack that had been prepared according to the commercial instructions and remain frozen for 48 h.

Throughout the study, three stool samples were reported as missed or not collected (one in HT and two in LT). In HT, two stool samples had a 24 delay in freezing. Samples were kept refrigerated during this delay.

#### 2.2.4. DNA extraction and sequencing (or sample processing and analysis)

Fecal samples were thawed immediately prior to processing and transferred on ice to EuroClone Biological safety cabinet. Samples were selected at random for processing. Samples were manually homogenized for 30 seconds before being processed in triplicate into separate tubes for 16sRNA analysis (0.2 g ± 0.05 g) and SCFA concentration (0.25 g ± 0.05 g). Once aliquoted, samples were frozen at −80°C until extraction.

SCFA analysis occurred at Science Analytical Facility at Edith Cowan University. SCFA concentrations were analyzed on Thermo Scientific GC-MS (ISQ) using a Thermo Scientific TG-Wax column (30 m × 0.25 mm × 0.25 μm) with a 7-point calibration. Extraction was completed in triplicate with two samples analyzed and the third analyzed as a spiked sample.

DNA was extracted using QIAamp® PowerFecal® Pro DNA kits (Qiagen). Mechanical lysis for 2 × 30 s on a TissueLyser II (Qiagen, the Netherlands) was included. Extractions were automated using a QIAcube Connect (Qiagen, the Netherlands) following the manufacturer’s instructions. DNA concentrations were estimated using the QIAxpert System (Qiagen, the Netherlands). PCR inhibitors were assessed by a 3-point dilution of each sample prior to quantitative polymerase chain reaction (qPCR) using same cycling conditions and primers as described below. For the amplicon PCR, two µL of the highest non-inhibited DNA dilution and 23 µL master mix were combined (14.45 µL ultrapure water, 2.5 µL reaction buffer (Applied Biosystems [ABI], USA), two µL magnesium chloride (ABI), one µL bovine serum albumin (Fisher Biotec, Aus), one µL 10 µM forward primer, one µL 10 µM reverse primer (Integrated DNA Technologies [IDT] Aus), 0.6 µL SYBR (Invitrogen, USA), 0.25 µL dNTPs (25 mm dATP, 25 mm dTTP, 25 mm dCTP, 25 mm dGTP; Astral Scientific, Aus), and 0.2 µL Ampli*Taq* Gold (ABI). Primers amplifying the V4 region of the 16S rRNA gene [24] were used. Both the forward primer (5’GTGCCAGCMGCCGCGGTAA’3) and reverse primer (5’GGACTACHVGGGTWTCTAAT’3) were combined with an eight-base multiplex identifier (MID) barcode. The combination of barcodes was unique for each sample. PCR amplifications were performed using a StepOnePlus Real-Time PCR System (Life Technologies, Thermo Scientific, USA). Samples were mini pooled in equal proportion based on ΔRn florescent values prior to quantification on the QIAxcel (Qiagen, the Netherlands). Mini pools were blended in equal molarity to form a library with a total volume of 200 μL. The library was then cleaned and concentrated to 30 μL using the QIAquick PCR purification kit (Qiagen, the Netherlands) prior to ligation with sequencing adaptors. The ligation protocol is based on the Lucigen NxSeq® AmpFREE Low DNA Library Kit following the end repair, A-tailing reaction and subsequent adaptor ligation reaction. At the completion of the adaptor ligation reaction, the library was cleaned using the QIAquick PCR purification kit and eluted in 60 μL. A fragment analysis of the ligated library was performed using the QIAxcel to determine ligation efficiency. The library was size selected to remove any residual adapter dimer using a Pippin Prep 2% agarose with ethidium bromide and external marker B cassette (Sage Science, USA). The size selected amplicon pool was purified a final time (QIAquick PCR purification, Qiagen) to remove ethidium bromide attained during size selection and eluted in 40 μL of EB buffer. The ligation library amplicon pool was serially diluted in EB buffer and quantified by qPCR against a standard 200 bp synthetic oligonucleotide (IDT) of known molarity. The qPCR result was used to approximate the amount of pooled ligation library required for successful clustering on the MiSeq Flowcell (Illumina, USA). The MiSeq sequencing set-up was carried out as per the manufacturer protocol. The amplicon library was bidirectional sequenced using a 500-cycle V2 reagent kit and a V2 Standard flowcell (Illumina, USA).

#### 2.2.5. Library preparation and sequencing

Sequence read quality was assessed with FastQC before demultiplexing and preprocessing by GHAPv2, an in-house tool. Cutadapt [25] was used for removal of all non-biological sequences. DADA2 [26] was then used for quality filtering, error correction, exact sequence variants (ASVs) picking. For this study, the forward sequences were trimmed at 165 bp and the reverse at 220 bp to maintain a constant read quality of >30 and the overlapping region between the two reads were >130 bp. A trained naïve bayes classifier (RDP) [27] then assigned nomenclature to ASVs against the curated Genome Taxonomy Database [28] of microbial reference sequences with minimum classification confidence of 50%.

#### 2.2.6. Data filtering

Data filtering removed all ASVs that occurred in less than 5% of samples or had a minimum of 100 counts of samples across the dataset. Any sequences that were unassigned at a phylum level were also removed. Sequences with the same species name with unidentified ASV were combined.

#### 2.2.7. Statistical analysis

Statistical analysis was completed using SPSS (version 29.0.0.0 (241), Primer 7 (version 7.0.23) and RStudio (2023.06.0 Build 421). To allow a comparison of timepoints, HT and LT, microbiome and SCFA data from one 24 h period was used to represent each timepoint. Data from the first 24 h period of each training load timepoint was selected. If this data was unavailable the second 24 h period was selected. All outcomes within a timepoint came from the same 24-h period i.e. microbiome composition, SCFA concentrations, stool frequency, and consistency. A combined stool score (CSS) was created by adding total number of samples with the consistency of each sample. Data normality was tested using Kolmogorov–Smirnov and Shaparo–Wilk tests in SPSS. Valeric acid at both timepoints, was normalized by applying a log transformation. SPSS was also used to provide descriptive statistics and complete paired *t*- tests. These were completed on demographic data, training load, macronutrient intake, diet quality, and short chain fatty acid concentrations.

Filtered counts were used for assessment of alpha diversity in Primer 7. Alpha diversity indices included total species, total individuals, species richness, Pielou’s Evenness, Shannon–Wiener Index, Simpson Index. Relative counts were calculated by dividing bacterial read count by total sample read counts. Relative counts were used for creation of PCO plots and Permanova analysis in Primer 7. Relative counts were transformed using fourth route with optimal contrast confirmed by shade plot. Bray Curtis Dissimilarity resemblance was selected because it copes well with zero inflated data. Permanova design template included subject number as a random factor nested in sex with sex and timepoint (HT and LT) as a fixed factor. Covariates were included via covariate worksheet. A central log ratio transformation was also completed on filtered counts and analysis re-run. No difference in outcomes was observed and relative data was reported.

Microbiome Multivariable Association with Linear Models were completed using MaAslin2 package in R. Filtered data with a CLR transformation was used to complete analysis. Sex and Timepoint were included as fixed factors and subject number as a random factor. Clustering of data into enterotypes was completed in R using tutorial provided at https://enterotype.embl.de/enterotypes.html#data. Data agglomerated at genus level was used to generate two main enterotypes. Characteristics were identified in Microsoft excel.

Distance-based Linear Model with marginal and sequential tests were run using total training stress score, total number of samples, percentage of diarrhea, weight, ADIcore, butyric acid and propionic acid. Marginal tests indicate how much each variable explains when all other variables are ignored. Sequential tests are included in specified order (training stress score, total number of samples, percentage of diarrhea, weight, ADIcore, butyric acid and propionic acid) it considers whether adding a variable contributes significantly the explained variation. Including sequential results provide further detail around relationship between variables. This analysis was completed in Primer 7, including all paired and complete data (*n* = 11 pairs). Regression analysis was conducted to identify relationship between variables and when variables were significantly correlated a representative variable was selected that had the most data available. Outcomes selected for inclusion in DIST-LM are therefore considered independent variables.

Regression analysis was completed using SPSS on key research outcomes and covariates within the scope of the research question. These variables were identified after initial paired t-test analysis confirming ADIcore and Stool frequency accompanied changes in training load. CSS was used instead of stool frequency and stool consistency (i.e. percent constipation, normal and percent diarrhea as it had a broader range of data and allowed zero results to be included (e.g. 0% diarrhea).

## 3. Results

### 3.1. Training load

Training duration (147% above LT) and TSS® (130% above LT) were, by design, greater during the 3 days of HT, as compared with LT (*p* < 0.001) (Table 1). HT was consistently higher than LT when summed across all modes of training including rowing, indoor rowing and cycling.

**Table 1.** Training load during periods of high load (HT) and Low load (LT).

	HT			LT		
Training Load	Mean	SD	n	Mean	SD	n
Training Duration (3 days)	7.31*	1.4	20	4.96	1.7	18
Training Stress Score® (3 days)	380.96*	98	20	249.36	92	18
Number of sessions (3 days)	7.4*	1.8	20	4.5	1.5	20

*p < 0.05 compared with LT.

### 3.2. Dietary intake

Total energy, protein, fat, carbohydrate intake was maintained between HT and LT. ADIcore was significantly higher during HT (55 ± 10) compared to LT (49 ± 9; t(15) = 2.78, *p =* 0.014; CI: 1.34 to 10.155) (Table 2).

**Table 2.** Dietary intake during periods of high load (HT) and Low load (LT).

	HT			LT		
Dietary Intake	Mean	SD	n	Mean	SD	n
Energy (MJ/d)	13.3	0.5	17	124.5	0.5	17
Protein (% energy)	17.6	2.5	17	16.0	3.0	17
Fat (% energy)	32.7	6.8	17	33.2	6.1	17
Carbohydrate (% energy)	47.7	6.7	17	47.9	5.3	17
Fibre (average g)	35.2	14.5	17	32.3	15.4	17
ADIcore	55*	10	16	49	9	16

*p < 0.05 compared with LT.

### 3.3. Stool frequency and consistency

Total number of stool samples in 48 h was greater during HT when compared with low training load (*n* = 18; 2.89 ± 1.32 vs 2.06 ± 1.30; t(17) = 2.83, *p =* 0.012; CI: 0.212 to 1.454). Similarly, minimum number of stool samples per day in 48 h (2 × 24 h collections) were greater during HT when compared with LT (*n* = 18; 1.11 ± 0.47 vs 0.67 ± 0.76; t (170 = 3.06, *p =* 0.007; CI: 0.138 to 0.752). Number of participants unable to produce a stool sample within 24 h during the 48 h collection period was 8% (*n* = 2) during HT and 47% (*n* = 9) during LT. No change was observed in stool consistency (Table 3).

**Table 3.** Stool frequency and consistency during periods of high load (HT) and Low load (LT).

	HT			LT		
Stool sample information	Mean	SD	n	Mean	SD	n
Number stools per day	2.89*	1.32	18	2.06	1.3	18
Minimum stools per day	1.11*	0.47	18	0.67	0.76	18
Maximum stools per day	1.78	0.94	18	1.39	0.69	18
Combined stool score (CSS)	5.75	3.64	16	4.68	2.35	16
Constipation – BSC^a^ 1–2 (%)	20.8	40.1	16	11.5	27.7	16
Normal – BSC^a^ 3–5 (%)	64.5	46.2	16	72.9	40.6	16
Diarrhoea – BSC^a^ 6–7 (%)	14.5	3.4	16	3.1	12.5	16
Participants unable to produce stool sample n (%)	2 (8)			9 (47)		

*p < 0.05 compared with LT.

^a^BSC Bristol Stool Chart.

### 3.4. Fecal sample characteristics

**Figure 1.**
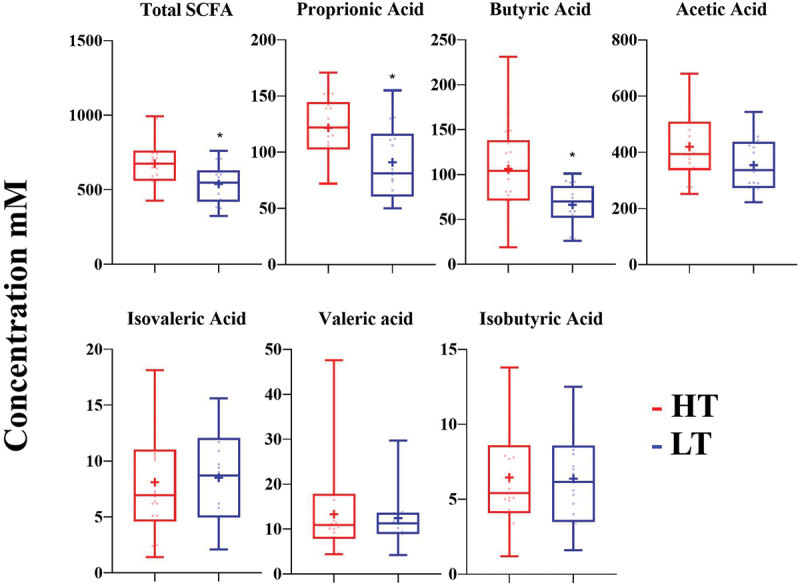
Participant SCFA concentrations during periods of high load (HT) and Low load (LT). All data points shown on plot, whiskers set at minimum and maximum values, box set at 25^th^ and 75^th^ percentiles, cross at mean, line at median. *p<.05 compared with HT.

Total short chain fatty acid (*n* = 17; t(16) = 2.65, *p =* 0.021; CI 20.12 to 212.34), propionic acid (*n* = 17; t(16) = 3.09, *p =* 0.07; CI 9.18 to 49.39) and butyric acid concentrations (*n* = 17; t(16) = 3.09, *p =* 0.07; CI 9.18 to 49.39) were greater during HT, compared with LT. Acetic, isobutyric, iso valeric and valeric acid concentrations were not statistically different between HT and LT (Figure 1).

Shannon-Weiner index was greater during HT, compared with LT (*n* = 16; t(15) = −2.98, *p =* 0.09, CI = −0.409 to −0.681) (Table 4). Shannon-Weiner index remained greater when the Euclidean distances were compared using a Permanova, including sex as a factor and athlete diet quality index (ADIcore) as a covariate (*p =* 0.042) and with total number of stool samples is used as a covariate (*p =* 0.0125). When combines stool score CSS is included as a covariate instead of total stool number there is no longer a difference between HT and LT (*p =* 0.542).

**Table 4.** Alpha diversity indices from fecal samples during periods of high load (HT) and Low load (LT).

	HT	LT
Total species	201 (± 42)	222 (± 47)
Total Individuals	39282 (± 14291)	43022 (± 9562)
Margalef’s index (richness)	19.05 (± 4.09)	20.71 (± 4.33)
Pielou’s Evenness	0.65489 (± 0.05544)	0.68300 (± 0.06275)
Shannon Index	3.4613 (± 0.3610)*	3.6709 (± 0.3474)
Simpson Index	0.91129 (± 0.04236)	0.92862 (± 0.04496)

*p < 0.05 compared with LT.

**Figure 2.**
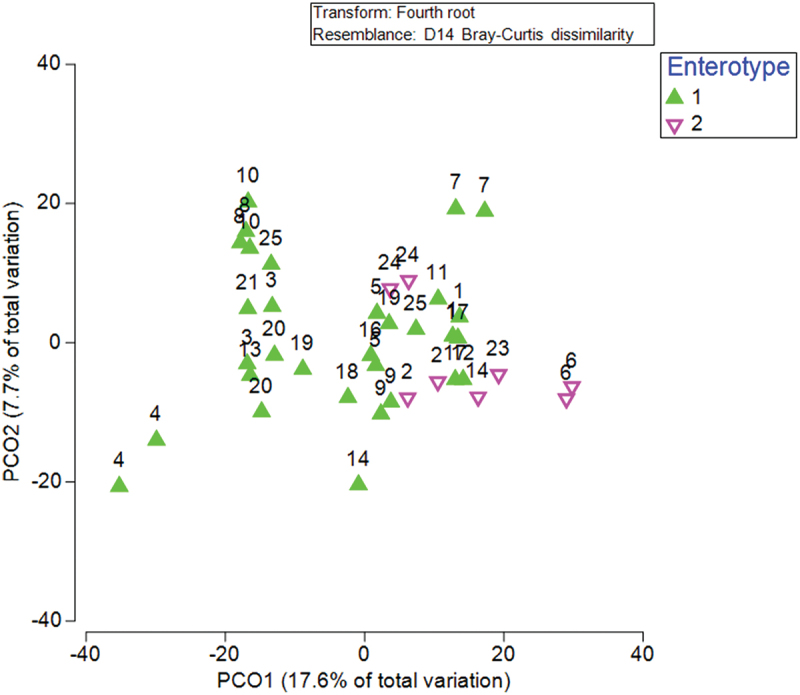
Principal coordinated analysis showing subject number and enterotype. Enterotype during both HT and LT are displayed.

**Figure 3.**
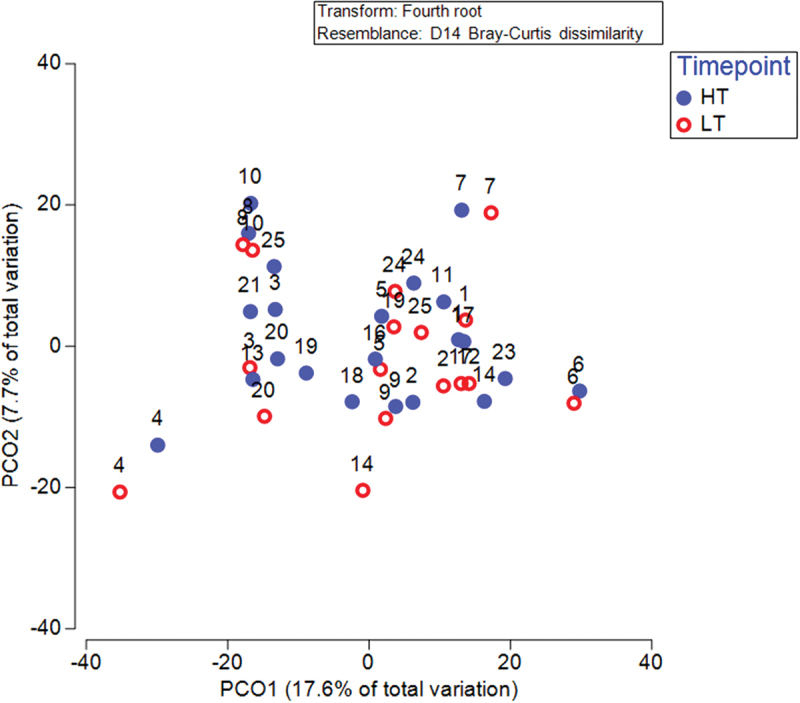
Principal coordinated analysis of beta diversity highlighting shifts between HT and LT.

**Figure 4.**
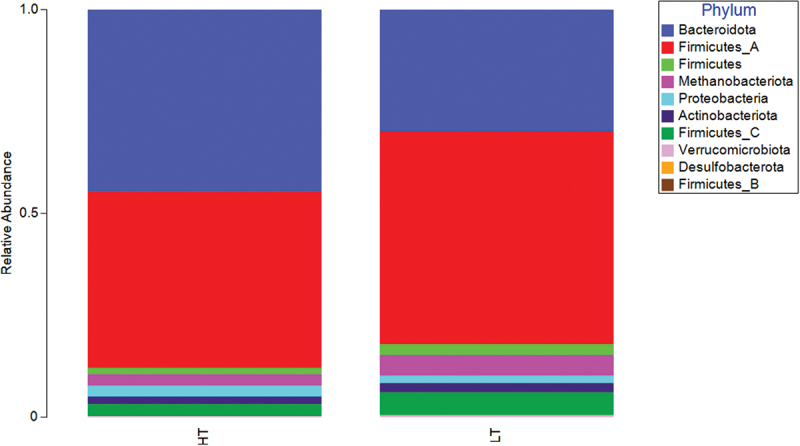
Relative abundance at phylum level for HT and LT.

Participants belonged to two main enterotypes, *Prevotella* dominant (Enterotype 2) or *Bacteroides/Rumicaccoae* dominant (Enterotype 1). These remained stable during HT and LT timepoint with only 1 participant (S14) shifting enterotype (Figure 2). Beta diversity was significantly different between HT and LT when comparing Bray Curtis dissimilarities and including sex as a factor (*p =* 0.047). Beta diversity was not different between HT and LT when ADIcore (*p =* 0.906) or number of stools per day (*p =* 0.75), were included as covariates (Figure 3). *Bacteroidota* were higher during HT compared to LT (corrected *p* = 0.039) and Firmicutes A trended lower during HT compared to LT (corrected *p =* 0.094) (Figure 4). The ratio of Firmicutes to *Bacteroidota* was lower during HT when compared with LT (*n* = 16, 1.31 ± 1.19 vs 4.29 ± 3.88, t(15) = −3.44, *p* = 0.04, CI = −4.82 to −1.13).

During HT, seven environmental factors (ADIcore, TSS, Diarrhoea%, Body weight, Butyric acid and Propionic acid, Total number of stool samples) explained 59.6% of the microbial variation with 40.4% of the variation remaining unexplained. ADIcore contributed to 12.2% of explained variation in the microbiome (*p =* 0.01), sequential tests indicate that diet quality, percentage of samples as diarrhea and weight contribute to 30.7% of the explained microbial variation. While training stress score only contributed to 7.5% of the explained microbial variation (Table 5). During LT, TSS makes the largest contribution (11.2%) to the explained microbial variation. When combined with total number of samples and percentage number of samples as diarrhea these three factors together explain 33.6% of the variation. ADIcore continues to contribute to explainable variation (11.2%) but is no longer significant. Short chain fatty acids, propionic (HT 7.6% and LT 8.3%) and butyric (HT 7.8% and LT 8.5%) did not significantly contribute to the variation in either timepoint (Table 5).

**Table 5.** Distance-based multivariate multiple regression of seven environmental factors during periods of high load (HT) and Low load (LT).

	Marginal Test			Sequential Test		
Variable	P	Prop.	P		Prop.	Cumul (%)
HT						
ADIcore	0.01*	0.122	0.02*		0.122	12.2
Diarrhoea – BSC 6–7 (%)	0.29	0.092	0.07		0.103	22.5
Body weight	0.23	0.085	0.21		0.082	30.7
Butyric acid	0.43	0.076	0.31		0.078	38.5
Propionic acid	0.59	0.071	0.34		0.076	46.2
Total training stress score	0.52	0.074	0.37		0.074	53.6
Total number of samples	0.06	0.103	0.58		0.060	59.6
LT						
Total training stress score	0.15	0.112	0.15		0.112	11.2
Total number of samples	0.71	0.076	0.09		0.121	23.3
Diarrhoea – BSC^a^ 6–7 (%)	0.50	0.084	0.23		0.104	33.6
Body weight	0.42	0.089	0.27		0.096	43.2
ADICore	0.29	0.097	0.09		0.115	54.8
Butyric acid	0.69	0.078	0.36		0.085	63.3
Propionic acid	0.20	0.104	0.37		0.083	71.5

*p < 0.05.

^a^Bristol Stool Chart.

The mean change in combined stool score (ΔCSS) from HT to LT showed a strong positive association with mean change in Shannon diversity (*r* = 0.562, *p =* 0.03) and mean change in relative abundance of *Bacteroides* (*r* = 0.562, *p =* 0.015) from HT to LT. No other significant correlations were observed (Table 6).

**Table 6.** Regression analysis on mean change from HT to LT for total SCFA concentrations, Shannon diversity, ADIcore, training duration, CSS and relative abundance of *Bacteroides.*

	r^2^	P	N
Δ SCFA vs Δ Shannon	−0.28	0.277	17
Δ SCFA vs Δ ADIcore	0.072	0.799	15
Δ SCFA vs Δ Duration	0.294	0.308	14
Δ SCFA vs Δ CSS^a^	0.272	0.29	17
Δ SCFA vs Δ Bacteroides	0.312	0.223	17
Δ Shannon vs Δ ADIcore	0.275	0.322	15
Δ Shannon vs Δ Duration	−0.148	0.614	14
Δ Shannon vs Δ CSS^a^	−.526*	0.03	17
Δ Shannon vs Δ Bacteroides	−0.389	0.123	17
Δ ADIcore vs Δ Duration	−0.403	0.194	12
Δ ADIcore vs Δ CSS^a^	−0.329	0.231	15
Δ ADIcore vs Δ Bacteroides	−0.461	0.083	15
Δ Duration vs Δ CSS^a^	0.237	0.414	14
Δ Duration vs Δ Bacteroides	0.19	0.516	14
Δ CSS^a^ vs Δ Bacteroides	.562*	0.015	18

*p <0.05.

^a^Combined stool score.

## 4. Discussion

The purpose of this study was to investigate the impact of training load on markers of gut health in trained athletes. To achieve this, we compared outcomes collected during a targeted high training phase (HT) with those collected during a low training (LT) phase or off-season. The main findings of the study were that LT compared with HT resulted in: i) a reduction in diet quality (ADIcore); ii) a decrease in stool frequency, and greater number of participants unable to produce a stool in 24 h; iii) decrease in total SCFA, propionic and butyric acid concentrations; iv) an increase in alpha, Shannon-Wiener diversity index; v) no change in overall enterotype but a shift in beta diversity with a reduction in the abundance of Bacteroidia and; vi) an increase in the ratio of Firmicutes to Bacteroidota due to a reduction in *Bacteroides* and the increase in Firmicutes A_Clostridia. Assessment of the driving factors behind changes in microbiome compositions indicate that training load, diet quality, and stool frequency are associated with changes in the gut microbiome.

Training load has an influence on athlete gut health markers with differences in SCFA concentration, the abundance of *Bacteroides* and alpha diversity observed. Importantly the difference in alpha diversity was observed when controlling for changes in diet quality, stool frequency and sex, indicating an independent influence of training load. The observed changes in this study are in line with previous research in athlete [6,9,11], non-athlete [25] and animal models [29] indicating that training load has an influence of the composition of the gut microbiota. Such associations are likely to be complex and involving multiple metabolic pathways. However, one interesting pathway is the potential role of the gut in the clearance of blood lactate resulting from the regular high volumes of anaerobic training observed at HT [11]. Indeed, such a hypothesis would explain the elevated butyrate and propionate concentrations observed in HT in the present study (Figure 1). There are a small number of microbes that are able to utilize lactate in the large intestine, but they play a key role in maintaining a healthy gut environment [30]. While the present study did not show changes in specific species of bacteria, it is likely that advanced cross feeding mechanisms exist to prevent lactate accumulation and ensure a healthy gut environment [31]. Furthermore, lactate produced in muscle is transported to the gut to be metabolized to butyrate and propionate [32], with these changes in total SCFA potentially resulting in an increase *Bacteroides*, and other butyrate, propionate and lactate utilizing bacterium [33]. Given the important role of lactate in anaerobic metabolism, the potential role of the gut in buffering lactate concentration during acute exercise is an interesting hypothesis that warrants further investigation.

It is important to highlight that in the present study participants were instructed to consume food *ad libitum* throughout the study with the deliberate intention of maximizing ecological validity of the study to provide an accurate understanding of changes in gut health through real-world changes in athlete training environments. As a result, lower training load was accompanied by a decline in diet quality and reduced stool frequency. It is, therefore, appropriate to consider the role of each of these factors when discussing the impact of training load on the composition of the gut microbiota. In the present study, a decline in diet quality using athlete diet index (ADIcore) [19] was observed when HT was compared to LT (Table 2). The relationship between ADIcore and changes in the composition gut microbiome is observed when ADIcore is included as a covariate in the analysis of beta diversity, changes between HT and LT are no longer significant (Figure 3). Furthermore, ADIcore explains a significant proportion (*p =* 0.01) of the variation in microbial composition at HT and explains 11% of the variation in microbial composition at LT (Table 5). These findings, in the athlete population, are novel and warrant further discussion.

The decline in ADIcore was observed during LT and was largely due to a reduction in the amount and variety of fruit and vegetables and an increase in the intake of takeout and discretionary foods. Although dietary changes are expected during an off season [15,19], it is interesting that in the present study, the decrease ADIcore during LT occurs while total energy, protein, carbohydrate and fiber intakes remain similar to those during HT (Table 2). This finding adds to literature in the general population [16,17,34] and athlete populations [6,8] suggesting that diet quality and not just quantity of nutrients impacts microbial composition and markers of gut health. The manipulation of diet quality to reduce gut health decline resulting from changes in training load would therefore be an interesting avenue for further research in the athlete population.

Concurrently, LT appeared to result in slower transit times when compared with HT. This is evidenced by a reduction in stool frequency and a greater number of participants experiencing no bowel action during LT, as compared with HT. The transit of food through the gastrointestinal tract, particularly a slowing of fecal matter through the colon, independently impacts the gut microbiota and microbial metabolism [17]. Increases in alpha diversity, reduction in SCFA concentrations and reduction in abundance of *Bacteroides* have been observed when transit times are slower [17,35,36]. In the present study, therefore, similar changes in the microbial composition between HT and LT, may be related to the observed reduction in stool frequency and increased incidence of no bowel movements (i.e. transit time) during LT. A positive correlation observed between the change in combined stool score (CSS) from HT and LT and change in Shannon and relative abundance of *Bacteroides* from HT to LT (Table 6) also suggests the change in stool frequency and consistency accompanying a reduction in training load is linked to observed increases in alpha diversity, reduction in SCFA concentrations and reduction in abundance of *Bacteroides*. This is of clinical relevance because monitoring transit time in athletes may be an efficient and effective tool for gaining meaningful insight into an athlete’s gut health. Further research examining the association between transit time and athlete gut health is warranted to explore the extent of this relationship.

The assessment of seven environmental factors (ADIcore, TSS, Diarrhoea%, Body weight, Butyric acid and Propionic acid, Total number of stool samples) indicates that an athlete’s microbiome is driven by a complex interaction between many factors (Table 5). Within the present study height and weight were collected on entry to the study, prior to HT, but was not reassessed prior to LT, an inclusion of this would have been beneficial. The addition other factors, not considered in this study (e.g. sleep and stress), may also play and their role in the composition of the microbiome and would be an interesting topic for future research.

Rigor surrounding collection of fecal samples and the inclusion of two consecutive 24 h whole stool collection provides confidence in microbial composition outcomes [37] but also increased participant burden with participants reporting challenges in completing all three components of the data collection (i.e. whole stool sample collection, food diary and training load including heart rate and accelerometer), during each training phase. As a result, number of participants with all outcomes are small limiting the power of some statistical analyses, particularly as a covariate in Permanova analysis, redundancy-based analysis and correlations. Given the degree of participant burden, we also decided not obtain details on female participants menstrual cycle. While it is plausible that menstrual cycle may impact results of the present study, such impacts are likely to be minimal [34]. While sex was used as a co-variate in this study, there was unfortunately insufficient statistical power to directly compare sexes.

## 5. Conclusion

As an athlete shifts from a phase of high training load to a low training load phase, changes in the composition of the gut microbiome occur. The present study indicates, the gut will be impacted by; i) the reduction of training load; ii) changes in dietary intake particularly a decrease diet quality and iii) slowing of transit time. This study also highlights an important relationship between each of these variables. The deliberate longitudinal design of this study targeting specific high and low training phases and allowing athletes to eat ad libitum throughout the study, had provided insight into real-world changes in athlete training environments and its relationship to the composition of the gut microbiome.

The role of transit time, SCFA concentrations, bacterial abundance and alpha diversity in athletic performance remains unclear but some potential mechanisms, including the role in lactate metabolism and gut pH modulation would likely impact athletic performance. Future studies that assess composition of microbiome and metabolic pathway activity would be beneficial to deepen insights into the relationship between training load, transit time, diet quality, and the composition of gut microbiome in highly trained athletes.
